# Paroxysmal sympathetic hyperactivity risk modeling based on transients in time series describing the autonomic nervous system and cerebral hemodynamics

**DOI:** 10.1007/s00701-025-06566-9

**Published:** 2025-05-30

**Authors:** Mikołaj Najda, Cyprian Mataczyński, Małgorzata Burzyńska, Magdalena Kasprowicz, Jarosław Kędziora, Emma Hammarlund, Eric P. Thelin, Agnieszka Uryga

**Affiliations:** 1 Institute of Data Science, Maastricht University, Maastricht, Limburg The Netherlands; 2 Faculty of Information and Communication Technology, Wroclaw University of Science and Technology, Wroclaw, Poland; 3 Faculty of Medicine, Clinical Department of Anesthesiology and Intensive Therapy, Wroclaw Medical University, Wroclaw, Poland; 4 Department of Biomedical Engineering, Wroclaw University of Science and Technology, Wybrzeze Wyspianskiego 27, 50-370 Wroclaw, Poland; 5 Medical Unit Neurology, Karolinska University Hospital, Stockholm, Sweden; 6 Department of Clinical Neuroscience, Karolinska Institutet, Stockholm, Sweden

**Keywords:** Autonomic nervous system, Cerebral hemodynamics, Traumatic brain injury, Paroxysmal sympathetic hyperactivity

## Abstract

**Purpose:**

Overstimulation of the autonomic nervous system (ANS) in the acute phase after traumatic brain injury (TBI) may lead to paroxysmal sympathetic hyperactivity (PSH) syndrome. This study aimed to investigate the impact of the relationship between ANS activity and cerebral hemodynamics on the development of PSH syndrome.

**Materials and methods:**

This retrospective study included 41 TBI patients admitted to Wroclaw University Hospital (Poland). Among them, 14 were classified as at risk for PSH based on the probabilistic Paroxysmal Sympathetic Hyperactivity Assessment Measure (PSH-AM), with 10 rated as ‘possible’ and 4 as ‘probable’. High-resolution neuromonitoring data from the first 72 h post-injury included intracranial pressure (ICP), pressure reactivity index (PRx), baroreflex sensitivity (BRS), arterial blood pressure (ABP), and heart rate (HR). The correlation between ANS activity and cerebral hemodynamics was quantified using the mean, standard deviation, and zero-crossing rate (ZCR) across sliding windows of 3, 6, 12, and 24 h. Logistic regression was used to model PSH risk.

**Results:**

The PSH risk model, including ZCR-based variability of ANS-cerebral hemodynamic correlations within a 3-h sliding window and adjusted by clinical metadata, achieved the highest performance (AUC 0.72 ± 0.27), outperforming the clinical metadata-only model (AUC 0.64 ± 0.18). Aggregated feature importance values indicated that the most predictive relationships were observed between HR–ICP and HR–PRx.

**Conclusions:**

Including the early post-injury interactions between ANS and cerebral hemodynamics in the clinical characteristics-based PSH risk model may improve its performance. Further studies in larger cohorts are necessary to validate these findings.

**Supplementary Information:**

The online version contains supplementary material available at 10.1007/s00701-025-06566-9.

## Background

Acute brain injury has been shown to cause neurogenic cardiac injury, which is independently associated with increased mortality and morbidity [66]. Traumatic brain injury (TBI) triggers a systemic surge in catecholamines driven by the central neuroendocrine axis, resulting in significantly increased sympathetic outflow and adrenal gland activation [30]. The mechanical insult caused by TBI can damage the insula and/or hypothalamus, causing an intense inflammatory response that activates and subsequently disrupts the autonomic nervous system (ANS) [59].

A sustained increase in sympathetic activity, marked by elevated circulating catecholamine levels, can persist for up to ten days and is thought to serve as a protective mechanism for maintaining cerebral perfusion in the presence of increased intracranial pressure (ICP) [30]. However, elevated catecholamine levels also induce several adverse effects, including systemic vasoconstriction, increased cardiac afterload, heightened myocardial workload, and elevated oxygen demand [59]. While sympathetic activation is an essential adaptive mechanism following brain injury, a prolonged hyperadrenergic state may adversely impact patient outcome [67].

In patients with severe TBI, the prevalence of dysautonomia or paroxysmal sympathetic hyperactivity (PSH) syndrome ranges from 8 to 33% [94]. PSH clinically manifests with symptoms such as tachycardia, noninfectious fever, hypertension, diaphoresis, or tachypnea [32]. The PSH Assessment Measure (PSH-AM) was proposed as an international consensus to define diagnostic criteria for PSH [5]. The PSH-AM scale comprises two components: the Clinical Feature Scale (CFS), which evaluates the severity of the core sympathetic and motor symptoms (tachycardia, tachypnea, hypertension, hyperthermia, sweating, and posturing during episodes), and the Diagnostic Likelihood Tool (DLT), which assesses the frequency and characteristics of PSH-compatible features [5]. Although the PSH-AM tool has demonstrated high sensitivity, it has limited specificity [73]. Moreover, identifying PSH syndrome can be biased by TBI-related complications such as seizures, sepsis, hypoxia, hypoglycemia, and traumatic pain [97]. As reported by previous studies, misdiagnosis or underrecognition remains a challenge in clinical practice [15, 35, 44]. Early diagnosis of PSH is also limited by the fact that certain diagnostic criteria are applicable only after specific clinical features have initially manifested [97].

Currently, increased attention is being given to multimodal monitoring in intensive care units (ICUs) and specialized neurocritical care units (NCCUs), enabling early identification of physiological disturbances and prevention or rapid treatment of secondary brain injuries [3, 41, 68]. In recent decades, biosignal analysis has evolved from focusing solely on average values to incorporating variability measures, such as dose and entropy, to enhance the understanding of physiology [87] [8]. Given that ANS activity dynamically responds to physiological changes, such as elevated ICP or decreased cerebral perfusion pressure (CPP) [23, 77], new metrics are needed to assess these temporal fluctuations.

Previous studies have highlighted the need for research on the relationship between ANS activity and cerebral hemodynamics [23, 26, 57]. However, no studies have comprehensively investigated the dynamic interplay between those two mechanisms in relation to PSH development during the early days following brain trauma. Adding information on regulatory interactions between the heart and brain may enhance PSH syndrome assessment. This study aimed to investigate how these interactions influence the development of PSH. We hypothesized that transient changes in the correlation between ANS activity and cerebral hemodynamics, when adjusted for clinical characteristics, could improve early identification of patients who may develop PSH syndrome.

## Materials and methods

### Study group

**Fig. 1:**
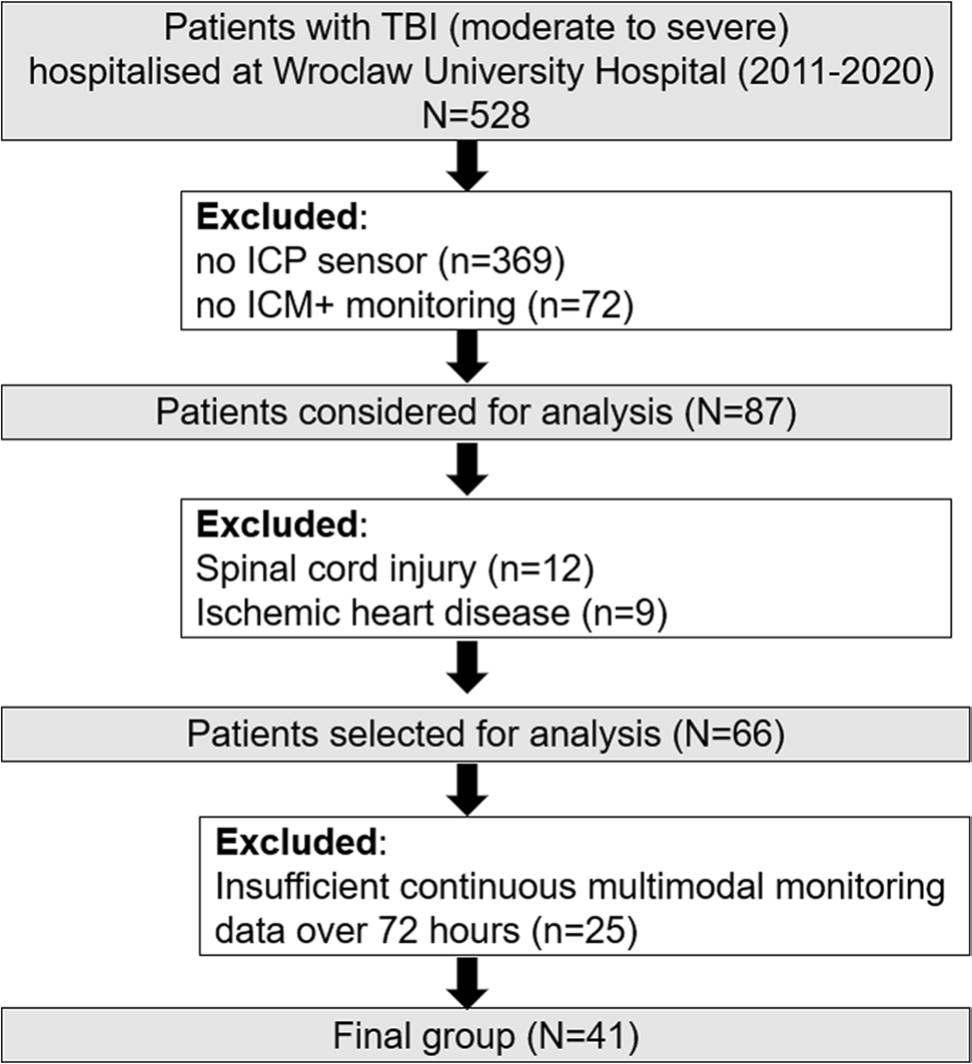
Flow chart. *Abbreviations:* TBI, traumatic brain injury; ICP, intracranial pressure; ICM +, intensive care monitoring software

This study retrospectively analyzed data from patients hospitalized with TBI at Wroclaw University Hospital, Poland (WUH) between 2011 and 2020. The retrospective analysis of data collected at WUH was approved by the Bioethics Committee of Wroclaw Medical University (approval KB-133/2023). The Bioethics Committee of Wroclaw Medical University waived the requirement for the investigator to obtain a signed informed consent form for all the subjects. The data were fully anonymized, and no data protection issues were involved. The inclusion criteria were as follows: patients aged over 18 years (adults), moderate to severe TBI defined by an admission Glasgow Coma Scale (GCS) score of 3 to 8 [38], with either an isolated head injury or predominant head injury in cases of multiorgan trauma, and the use of an ICP sensor. The exclusion criteria included preexisting ischemic heart disease that could interfere with ANS function, concurrent traumatic spinal cord injury due to its association with an increased risk of dysautonomia [85], poor-quality signal recordings, and late initiation (> 24 h posttrauma) of multimodal monitoring. At the preprocessing stage, multimodal monitoring data for each patient was evaluated to ensure that signals were continuous over a 72-h period, allowing for a maximum of 10% missing values within this window to confirm a robust dataset for further correlation analysis. A flow chart illustrating the study design is presented in Fig. 1. We acknowledge that our cohort was partially analyzed in a previous study, where we analyzed the relationship between the ANS and cerebral autoregulation [12].

### Patient management

Patients were treated according to the head injury treatment guidelines at the time of admission (Brain Trauma [9, 13]). After an initial evaluation and stabilization in the emergency department, guided by Advanced Trauma Life Support (ATLS) protocols [11] addressing airway, breathing, and circulation, patients were either admitted directly to the ICU or transferred postoperatively following urgent surgical intervention or the placement of an ICP sensor. Throughout the entire period, encompassing the first three days of the patient's ICU stay, all patients were sedated, intubated, and received mechanical ventilation. An ICP/CPP management algorithm was applied to maintain the ICP below 20 to 22 mmHg (in accordance with the guidelines in effect at the time of admission)(Brain Trauma [9, 13])), and the CPP was above 60 mmHg through therapeutic interventions when necessary. The initial neurological status of each patient was determined using the GCS upon admission to the hospital. The Injury Severity Scale (ISS) was used to quantify the severity of the injury according to the Berlin definition [34, 84]. The ISS is a composite score based on an Abbreviated Injury Scale (AIS) score ≥ 3 for two or more different body regions in conjunction with additional physiological parameters (age, consciousness, hypotension, coagulopathy, and acidosis), where ISS ≥ 16 refers to serious injuries. A standard assessment of acid‒base balance was conducted, involving repeated measurements of arterial blood gas and pH analysis (at least four times a day) using ABL800 FLEX Radiometer (Radiometer Medical ApS, Denmark). Metabolic acidosis was defined as a primary acid excess or base deficiency disorder [1]. Trauma-induced coagulopathy was defined as an abnormal hemostatic response secondary to trauma. Diagnosis was based on clinical evaluation and monitoring of hemostasis parameters, including traditional laboratory parameters such as the kaolin–kefalin time, prothrombin time/international normalized ratio, fibrinogen level, platelet count, antithrombin level and D-dimer level [20]. Blood morphology results obtained at admission were analyzed, including the hemoglobin (Hb) concentration, which can impact cerebral oxygenation optimization [6], and the white blood count (WBC), as TBI can induce an acute inflammatory response that may exacerbate secondary brain damage [31]. The Marshall Computed Tomography (CT) Classification [51] and Rotterdam CT score [48] were used to assess the severity of TBI on the basis of CT findings at admission. Data on posttraumatic changes observed on admission CT scans, including subdural hematoma (SDH), epidural hematoma (EDH), traumatic intracerebral hematoma (tICH), edema, diffuse axonal injury (DAI), and traumatic subarachnoid hemorrhage (tSAH), were collected. The outcome was assessed at hospital discharge using the Glasgow Outcome Scale (GOS) and dichotomized into unfavorable (1–3) and favorable (4–5) categories [38].

### Paroxysmal sympathetic hyperactivity risk assessment

All patients were monitored daily for general and neurological changes during their stay in the ICU. The PSH Assessment Measure (PSH-AM) clinical scoring was used to diagnose PSH [5]. This scale consists of two components: the Clinical Feature Scale (CFS), which identifies the intensity of cardinal features (heart rate, respiratory rate, systolic blood pressure, temperature, sweating, posturing during episodes), and the Diagnosis Likelihood Tool (DLT), which evaluates the likelihood of PSH presence. The total PSH-AM score is the sum of these two components (CSF + DLT). A PSH-AM score of less than 8 indicates that PSH is unlikely, a score of 8–16 suggests a possible diagnosis, and a score greater than 17 indicates that PSH is probable [55]. A detailed description of the PSH-AM scale is provided in the Supplementary material (Supplementary Table 1). In this study, based on the clinical course, two independent investigators assessed PSH syndrome using criteria defined in PSH-AM tool after excluding other causes (e.g., poorly controlled pain, infection, epileptic seizure). Because of the limited number of patients, we categorized patients as being at risk of PSH (PSH-AM score ≥ 8;‘possible’, ‘probable’) or not at risk of PSH (PSH-AM score < 8; ‘unlikely’-). This PSH risk category was subsequently used as a model outcome.

### Signal monitoring

Signal monitoring was initiated within the first 24 h after onset and performed continuously. We included data from the first three days (72 h) of multimodal monitoring. Although the most critical time frame for ANS dysfunction analysis has not yet been clearly defined, recent studies suggest that the first 72 h after TBI may be crucial [7, 19, 28, 72, 92, 93]. Arterial blood pressure (ABP) was measured invasively in the radial artery using a pressure transducer (Argon Standalone DTX Plus™, Argon Medical Devices Inc. Plano, TX, USA). The ICP was measured invasively using intraparenchymal probes (Codman MicroSensor ICP Transducer, Codman & Shurtleff, Randolph, MA, USA) inserted into the frontal cortex and the transducer was placed at the level of the foramen of Monro. Data were recorded at a sampling frequency of 200 Hz using the Intensive Care Monitor (ICM +) system (Cambridge Enterprise Ltd., Cambridge, UK). Initially, all artifacts were identified and manually removed from the raw data files in the ICM + software before proceeding with further analysis.

### Neuromonitoring parameters

Cerebral hemodynamics were characterized using ICP and pressure reactivity index (PRx). PRx, which assesses cerebrovascular reactivity, was defined as the Pearson linear correlation coefficient between slow-wave oscillations of ABP and ICP within a 5-min moving average window, updated every 10 s [17]. The ANS activity was assessed using mean ABP, mean HR, and baroreflex sensitivity (BRS). Heart rate (HR) was determined using Fast Fourier Transform as the frequency associated with the first harmonic of ABP (range: 40–140 beats/minute,0.67–2.33 Hz). BRS was estimated in the time domain using the cross-correlation method [89, 90] as the slope of the regression line between 10-s segments of the systolic peak-to-peak interval and the corresponding systolic pressure time series derived from the ABP signal. The cross-correlation function was used to obtain the maximum correlation coefficient, considering the unknown time shift between the series within the range of 0–5 s. The mean values of all the neuromonitoring parameters were calculated via ICM + software by averaging the values in the 60-s time window.

### Rolling window correlation analysis

Signal correlations were calculated using a rolling window approach to track the time-dependent relationships between the ANS and cerebral hemodynamics. Missing values were treated by linear interpolation when up to 10 consecutive data points were missing. For each signal pair, including ANS metrics (HR, ABP, BRS) and cerebral hemodynamics (ICP, PRx), rolling Pearson’s correlations were calculated over sliding windows of 3, 6, 12, and 24 h, allowing for a multiscale examination of signal relationships. Three variability metrics were extracted from the values within each window and aggregated: the mean of the correlation values (MEAN), standard deviation (STD), which measures the variability of the correlation values, and zero-crossing rate (ZCR), which quantifies how frequently the correlation changes sign (Eq. 1):

1 $${\text{ZCR}}=\frac{1}{N-1}{\sum }_{n=1}^{N-1}\left|{\text{sgn}}\left(corr\left[n\right]\right)-{\text{sgn}}\left(corr\left[n-1\right]\right)\right|$$

where *N* is the total number of rolling correlation samples, *corr[n]* is the rolling correlation value at sample number *n* in {0…, N}, and *sgn* is the signum function that extracts the sign of a real number. Its value has been used in speech recognition, analysis of electroencephalogram features for comprehensive brain analysis, or the complexity of the heartbeat signal [62].

### Dataset design

**Fig. 2:**
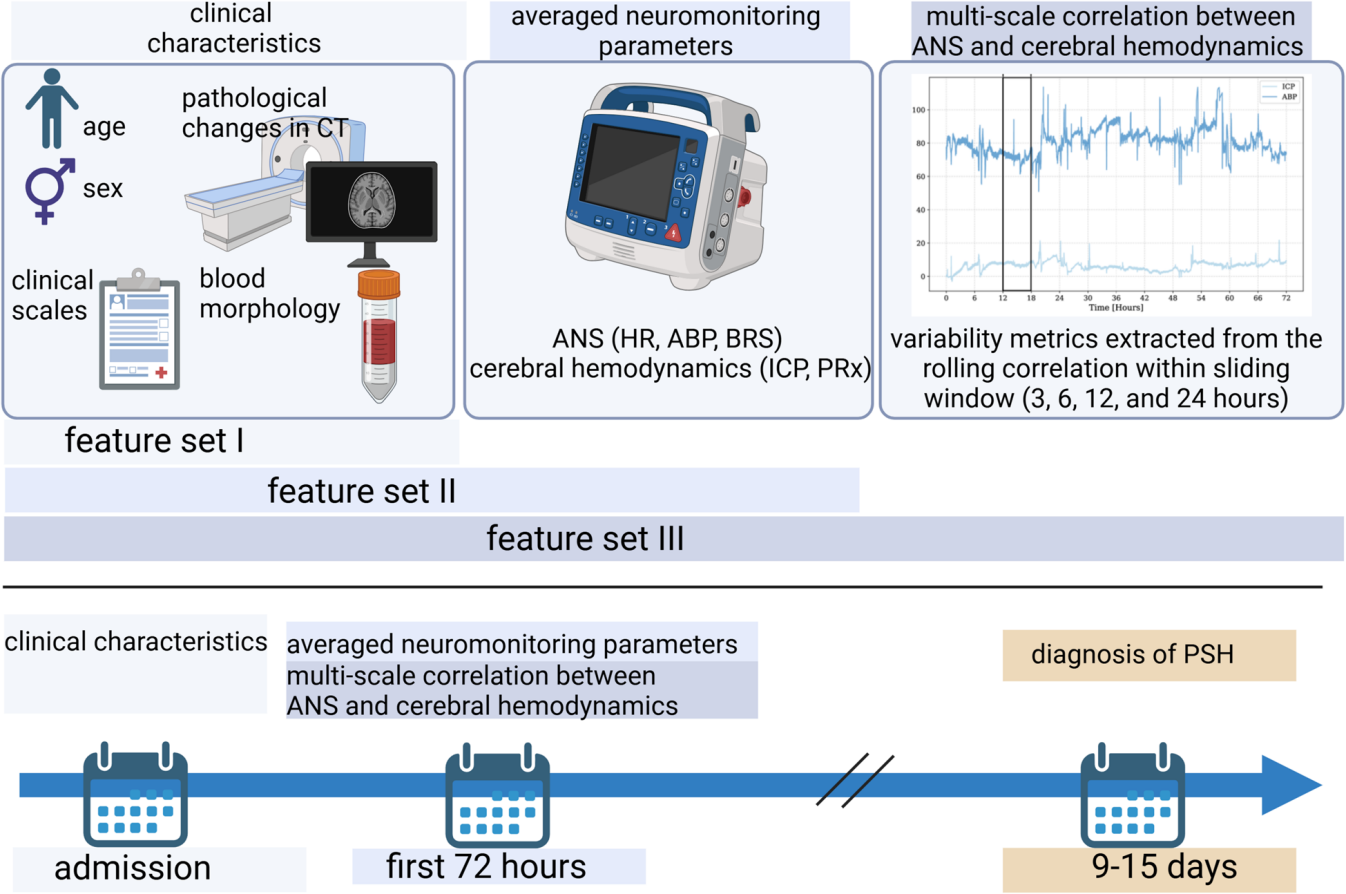
Study design. This retrospective study included 41 traumatic brain injury (TBI) patients hospitalized at Wroclaw University Hospital (Poland), of whom 14 were diagnosed as being at risk for paroxysmal sympathetic hyperactivity (PSH) using the Clinical PSH Assessment Measure (PSH-AM) between days nine and fifteen post-injury. PSH risk modeling was performed using clinical metadata, averaged neuromonitoring parameters, and metrics describing the variability of correlation between autonomic nervous system (ANS) and cerebral hemodynamics parameters over sliding windows of 3, 6, 12, and 24 h. Created at https://BioRender.com

Three different feature sets were used to model PSH risk. Feature set I included the following clinical metadata: general characteristics (age, sex [male]), GCS sum score, ISS), type of trauma (isolated head injury or predominant head injury in multiorgan injury), basic blood morphology results obtained at admission (Hb, WBC), and the presence of post-traumatic changes on admission CT (SDH, EDH, tICH, edema, DAI, and tSAH). Feature set II included feature set I and the mean of neuromonitoring parameters (ABP, HR, BRS, ICP, and PRx) averaged over the first 72 h. Feature set III included feature set II and one type of aggregated variability metric (ZCR, STD, MEAN) extracted from the rolling correlation between ANS metrics (BRS, ABP, HR) and cerebral hemodynamics (ICP, PRx) from the first 72 h within one of the following sliding windows of 3, 6, 12, and 24 h. The study design is presented in Fig. 2.

#### Statistical analysis

The Shapiro- Wilk test was used to determine whether the data were normally distributed. Differences in median values, categorized by dichotomized criteria, were tested using the Mann–Whitney U test for numeric data or the χ^2^ test (Fisher’s exact test) for nonnumerical data. The significance level of all tests was set at 0.05. The results are presented as the median value ± interquartile range (IQR) unless otherwise indicated. The analysis follows the Transparent Reporting of a multivariable prediction model for Individual Prognosis Or Diagnosis (TRIPOD) guidelines (Supplementary materials). A logistic regression model was used for the binary classification to identify patients at risk of PSH syndrome. The model was constructed using the following regularization strength (2*N*, *N*, 1, 1/*N*, and 1/(2*N*)), where *N* represents the number of instances. Class weighting was explored in both balanced and unbalanced settings. To ensure generalizability, stratified five-fold cross-validation was applied, maintaining a consistent class distribution across training and validation sets. Each fold used 80% of the data for training and 20% for validation. The primary evaluation metrics were the area under the receiver operating characteristic (ROC) curve (AUC) and accuracy (ACC). In the stratified five-fold cross-validation, the logistic regression model parameters were as follows: features set I – regularization strength of 41, balanced class weighting; features set II – regularization strength of 0.025, unbalanced class weighting; features set III – regularization strength of 82, unbalanced class weighting. To assess the importance of each variable, feature importance and SHapley Additive exPlanations (SHAP) values were used. Feature importance was defined as the absolute values of the regression coefficients. SHAP values provided a model-agnostic explanation by distributing impact across all features, reflecting each feature’s influence compared to its baseline value [45]. The temporal profile of significance for ANS-cerebral hemodynamic correlation was derived from aggregated absolute values obtained separately from the feature importance and SHAP across all models in the stratified five-fold cross-validation, based on the feature set with the highest scores. These values were rescaled using min–max normalization (range 0 to 1). The most influential timespan was identified based on values exceeding the threshold defined by the mean of normalized aggregated feature importance values. Statistical analysis was conducted using STATISTICA 13 (Tibco, Palo Alto, USA) and Python 3.10.14. The source code is available at https://github.com/AUTOMATIC-BRAIN-ANS/PSH_Probability.

## Results

### Patient characteristics

From 2011–2020, a total of 528 patients diagnosed with TBI were admitted to the ICU at Wroclaw University Hospital and assessed for potential study participation. Among them, 41 (76% men) met the inclusion criteria and were included in the study (Fig. 1). The median (upper-lower quartile) age was 33 (28–50) years. The group was classified as having severe TBI, with a median injury severity score (ISS) of 25 (20–34, AIS head ≥ 3) and a median total GCS score of 6 (4–8) with motor subscores of 4 (2–5). The main causes of brain trauma were vehicle collisions (21 (51%)) and other nonintentional injury (12 (29%)). Within the study cohort, 39% of patients (n = 16) presented with isolated severe TBI, characterized by a TBI AIS score of ≥ 3, other body region AIS of ≤ 2, and a GCS below 8. PSH syndrome was classified as probable in 4 (10%) subjects, possible in 10 (24%), and unlikely in 27 (66%). Clinical manifestations of PSH occurred between days nine and fifteen of ICU treatment. Patients were dichotomized into two groups: those at risk of PSH (n = 14, with 10 having scores between 8–16 on the PSH-AM scale and 4 having scores > 17) and those not at risk of PSH (n = 27, with PSH-AM scores < 8). The clinical characteristics of the patients concerning PSH syndrome assessment are presented in Table 1.

**Table 1:** Clinical characteristics of patients with severe traumatic brain injury (TBI) stratified by the risk of paroxysmal sympathetic hyperactivity (PSH). Data are presented as the median (upper-lower quartile) or number of subjects (% of the subgroup)

Parameter	Total cohort (*n* = 41)	At risk of PSH (*n* = 14)	None at risk of PSH (*n* = 27)	*p*-value
Age	33 (28–50)	33 (28–40)	38 (28–52)	0.856
Sex (Female)	10 (24%)	5 (36%)	5 (19%)	0.232
Cause of injury:				
Road traffic incident	21 (51%)	7 (50%)	14 (52%)	0.569
Incidental fall	4 (10%)	2 (14%)	2 (7%)	
Other nonintentional injury	12 (29%)	4 (29%)	8 (30%)	
Violence/Assault	3 (7%)	0	3 (11%)	
Other	1 (3%)	1 (7%)	0	
Pupils reactivity:				
Bilateral unreactive	2 (4%)	1 (7%)	1 (4%)	0.477
Unilateral unreactive	8 (20%)	4 (29%)	4 (15%)	
Bilateral reactive	31 (76%)	9 (64%)	22 (81%)	
ISS scale	25 (20–34)	24 (20–34)	25 (18–41)	0.817
GCS at admission	6 (4–8)	6 (4–8)	6 (4–8)	0.856
GCS motor at admission	4 (2–5)	4 (2–4)	4 (2–5)	0.307
CT characteristic:				
Marshall CT Score	3 (2–4)	3	3 (2–4)	0.753
Rotterdam CT Score	3 (2–4)	3 (2–4)	3 (2–4)	0.568
Edema	28 (68%)	10 (71%)	18 (67%)	0.756
SDH	19 (46%)	8 (57%)	11 (41%)	0.318
EDH	2 (5%)	0	2 (7%)	0.423
tICH	9 (22%)	1 (7%)	8 (30%)	0.102
DAI	33 (80%)	11 (79%)	22 (81%)	0.823
tSAH	22 (54%)	6 (43%)	16 (59%)	0.318
WBC [10^3^/ul]	16.0 (13.0–20.0)	20.3 (15.8–21.9)	14.2 (10.1–17.4)	**0.004**
Hb [gdl]	13.1 (11.3–14.4)	13.2 (11.3–14.4)	13.1 (11.0–14.7)	0.754
Neuromonitoring:				
ABP [mm Hg]	84 (80–88)	87 (80–88)	82 (79–88)	0.454
HR [bpm]	78 (70–86)	78 (70–89)	76 (70–86)	0.881
BRS [ms/mm Hg]	6.8 (3.9–9.8)	6.9 (4.5–8.5)	6.8 (3.6–10.6)	0.694
ICP [mm Hg]	11 (6–13)	12 (9–13)	10 (6–13)	0.522
PRx [a.u.]	0.05 (0.00–0.17)	0.06 (0.02–0.22)	0.05 (−0.04–0.16)	0.391
Outcome at hospital discharge:				
Hospital LOS [days]	27 (16–41)	30 (15–47)	22 (16–41)	0.634
Mortality	7 (17%)	2 (14%)	5 (19%)	0.733
GOS	3 (2–3)	3 (2–3)	3 (3–4)	0.281
Unfavorable, GOS 1–3	33 (80%)	13 (93%)	20 (74%)	0.153
Favorable, GOS 4–5	8 (20%)	1 (7%)	7 (26%)	

*Abbreviations: ISS* Injury Severity Score, *GCS* Glasgow Coma Scale, *CT* Computed tomography, *SDH* Subdural hematoma, *EDH* Epidural hematoma, *tICH* Traumatic intracerebral hematoma, *DAI* Diffuse axonal injury, *tSAH* Traumatic subarachnoid hemorrhage, *GOS* Glasgow Outcome Scale, *LOS* Length of stay, *PSH* Paroxysmal sympathetic hyperactivity, *WBC* White blood cell count, *Hb* Hemoglobin, *ABP* Arterial blood pressure, *HR* Heart rate, *BRS* Baroreflex sensitivity, *ICP* Intracranial pressure, *PRx* Pressure reactivity index, p-refers to the Mann‒Whitney U test or CHI^2^ test

### Feature set I: clinical metadata

**Fig. 3:**
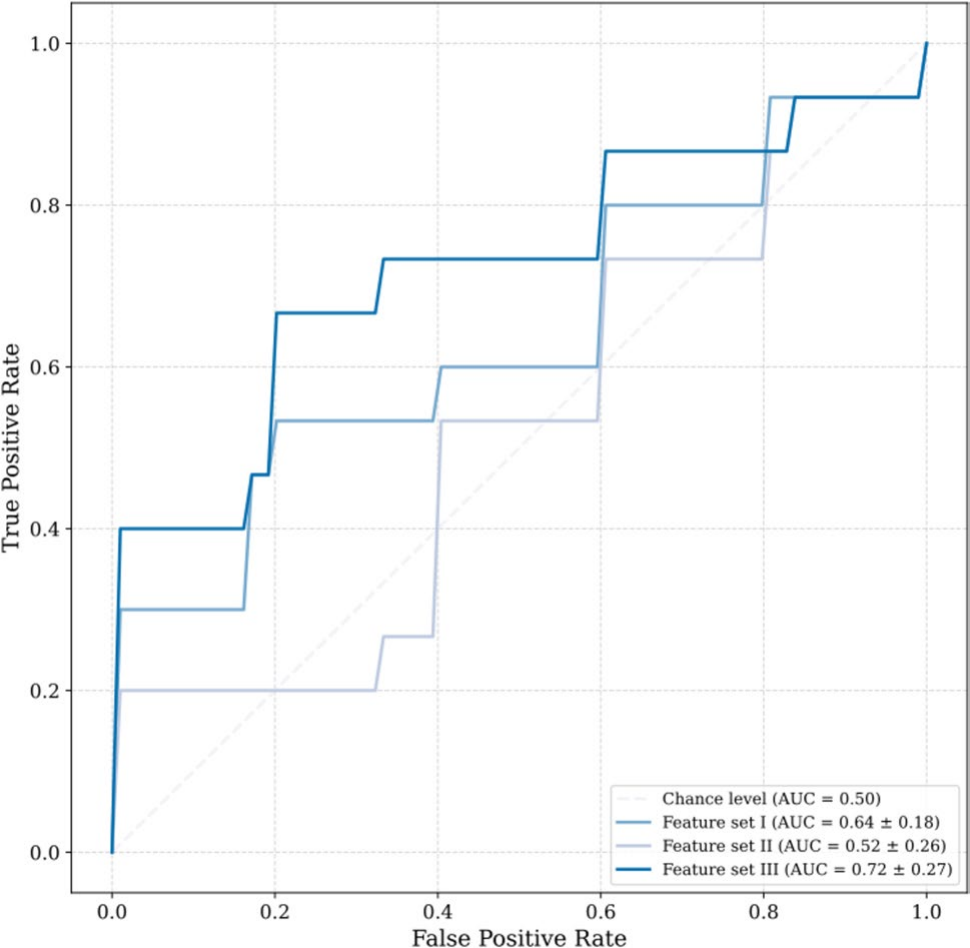
The discriminative ability of feature sets to differentiate patients at risk of paroxysmal sympathetic hyperactivity (PSH) from those not at risk is presented as the area under the receiver operating characteristic (ROC) curve (AUC). Feature set III, which includes the variability of the correlation between the autonomic nervous system and cerebral hemodynamics, achieved the highest score

A comparison of the clinical characteristics regarding PSH risk is presented in Table 1. There were no significant differences in age, sex, clinical scales, or CT findings between patients at risk of PSH and those not at risk of PSH (Table 1), except for basic blood cell analysis. The WBC count was significantly greater in patients at risk of PSH (20.3 (15.8–21.9) × 10^3^/µl) than in patients not at risk of PSH (14.2 (10.1–17.4) × 10^3^/µl), p = 0.004. The best logistic regression model for PSH risk based on the clinical metadata achieved an ROC AUC of 0.64 ± 0.18 and an ACC of 0.56 ± 0.17. The ROC curve is presented in Fig. 3.

### Feature set II: clinical metadata and averaged neuromonitoring parameters

A comparison of the average values of the neuromonitoring parameters is presented in Table 1. We found no significant differences in ABP, HR, BRS, ICP, or PRx between patients classified as at risk and those not at risk of PSH. The best logistic regression model for PSH risk based on the clinical metadata and averaged neuromonitoring parameters achieved an ROC AUC of 0.52 ± 0.26 and an ACC of 0.66 ± 0.05. The ROC curve is presented in Fig. 3.

### Feature set III: clinical metadata, averaged neuromonitoring parameters, and variability of rolling correlation

A grid search procedure was performed to choose the best setup of variability metrics (ZCR, STD, MEAN) extracted from the rolling correlation values within each of the following sliding windows: 3, 6, 12, and 24 h. The ZCR over a sliding window of 3 h showed the most significant enhancement in model performance when evaluated against the STD and MEAN, as shown in Table 2. The best logistic regression model for PSH risk based on clinical metadata, averaged neuromonitoring parameters, and ZCR over a sliding window of 3 h achieved an ROC AUC of 0.72 ± 0.27 and an ACC of 0.64 ± 0.19. The ROC curve is presented in Fig. 3.

**Table 2:** The best area under the receiver operating characteristic (ROC) curve (AUC) and accuracy of the logistic regression model for assessing the risk of paroxysmal sympathetic hyperactivity (PSH) for each window size using feature set III. Feature set III was based on clinical metadata, averaged neuromonitoring parameters, and a single type of aggregated variability metric extracted from the rolling correlation values between the autonomic nervous system (ANS) and cerebral hemodynamic parameters over sliding windows. A grid search procedure was performed to select the best variability metric—the zero-crossing rate (ZCR), standard deviation (STD), or mean value (MEAN)—which was calculated within sliding windows of 3, 6, 12, and 24 h. Only the variability metric that achieves the highest AUC score is presented for each window size. The AUC and accuracy values are presented as the means and standard derivations

	Window [hours]			
	**3**	**6**	**12**	**24**
Variability metric	ZCR	STD	STD	STD
AUC	0.72 ± 0.27	0.63 ± 0.19	0.66 ± 0.18	0.61 ± 0.24
Accuracy	0.64 ± 0.19	0.63 ± 0.15	0.66 ± 0.20	0.54 ± 0.15

*Abbreviations*: *ZCR* Zero-crossing rate, quantifying how frequently the correlation changes sign, *STD* Standard deviation measuring the variability of the correlation value

### The temporal profile of significance for ANS-cerebral hemodynamics correlation

**Fig. 4:**
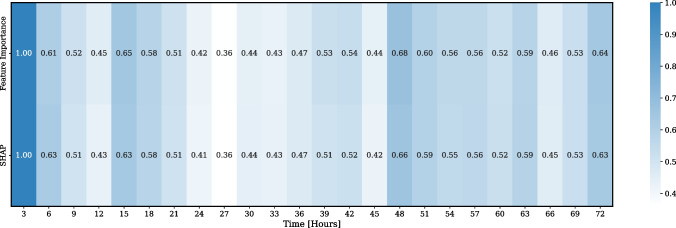
The time span of the significance of the autonomic nervous system–cerebral hemodynamics correlation, measured as the zero-crossing rate (ZCR) over a 3-h sliding window, within the paroxysmal sympathetic hyperactivity (PSH) risk model. Feature importance and SHAP absolute values were aggregated across all the models in the stratified five-fold cross-validation. These values were then rescaled using min–max normalization to a range between 0 and 1. The mean aggregated values were 0.55 ± 0.12 for feature importance and 0.54 ± 0.12 for SHAP

The temporal profile of significance of the ANS-cerebral hemodynamics correlation, measured as the ZCR over a 3-h sliding window in the PSH risk model, is presented in Fig. 4. The mean aggregated values were 0.55 ± 0.12 for feature importance and 0.54 ± 0.12 for SHAP. The most influential time period was identified as the first three hours of monitoring.

### Key factors in PSH risk modeling

**Fig. 5:**
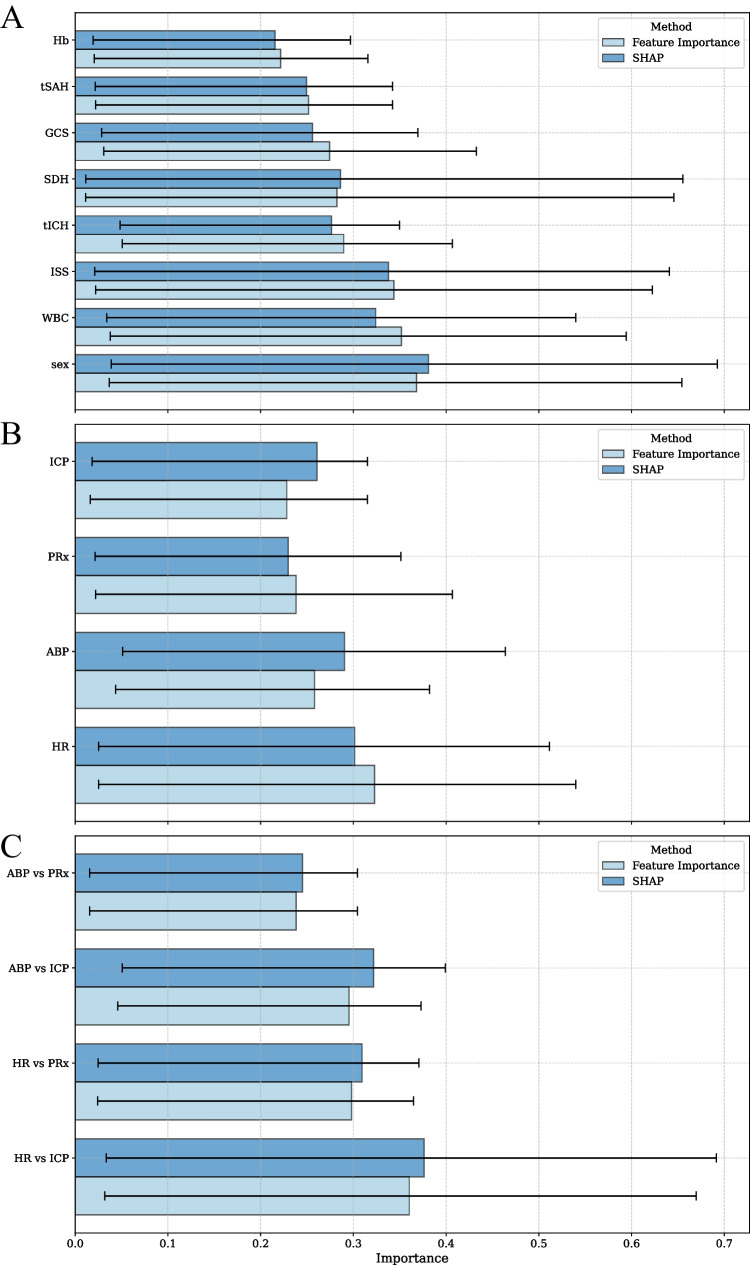
The most important features for assessing paroxysmal sympathetic hyperactivity (PSH) risk were determined using feature importance and SHAP methods. The results were aggregated for the logistic regression models based on feature set III. The importance analysis of the correlation between the autonomic nervous system (ANS) and cerebral hemodynamics metrics was provided using zero-crossing rate (ZCR) within the first 3 h. Panel **A** presents clinical metadata, Panel **B** averaged neuromonitoring parameters, and Panel **C** variability of rolling correlations between ANS metrics and cerebral hemodynamics. *Abbreviations*: Hb, hemoglobin; tSAH, traumatic subarachnoid hemorrhage; GCS, Glasgow Coma Scale; SDH, subdural hematoma; tICH, traumatic intracerebral hematoma; ISS, injury severity score; WBC, white blood cell; HR, heart rate; ABP, arterial blood pressure; ICP, intracranial pressure; PRx, pressure reactivity index

To identify the most important features in PSH risk prediction, we employed two methods: feature importance and SHAP. The results for each logistic regression model, based on the parameter permutations for Feature set III in the stratified five-fold cross-validation, were aggregated within summation and normalized. The importance analysis of the correlation between the ANS and cerebral hemodynamics metrics was provided using ZCR within the first three hours, as this period was found to be the most influential (Fig. 4). The results of the feature importance analysis are presented in Fig. 5 A-C. Among the clinical metadata, Hb, WBC, ISS, GCS, sex, and selected CT pathologies (tSAH, SDH, tICH) were identified as the most significant factors for PSH risk (Fig. 5A). For neuromonitoring parameters, HR and ABP were identified as the most influential parameters for risk of PSH development (Fig. 5B). The correlations between HR–PRx and HR–ICP were found to be the most influential factors for PSH risk (Fig. 5C). The analysis of the mean ± standard deviation of the aggregated logistic regression coefficients for ZCR_HR-PRx_ (0.30 ± 0.32) and ZCR_HR-ICP_ (0.36 ± 0.33) indicates that greater instability was associated with a higher likelihood of PSH. Both feature importance analysis methods were consistent and yielded similar results.

## Discussion

In this study, we introduced an integrative approach to modeling PSH syndrome occurrence in the early phase after TBI by analyzing the relationship between ANS metrics, ICP, and cerebrovascular reactivity within the first 72 h. Our findings suggest that adding the assessment of the correlation between ANS activity and cerebral hemodynamics to the clinical metadata-based PSH risk model may improve its performance. The relationships between HR–ICP and HR–PRx were found to be the most significant for PSH risk assessment.

We proposed a new approach to analyze the correlation between ANS activity and cerebral hemodynamics based on high-resolution neuroparameters. This association was characterized not only by its mean value but also by its standard deviation, which captures variability, and by the zero-crossing rate, which indirectly reflects changes in the direction of correlation within a rolling time window. To our knowledge, this is the first study to investigate the utility of transients between the ANS and cerebral hemodynamics for PSH risk assessment. In most current studies, this association has been assessed using simple methods, such as averaging indices over the total recording period [58, 77]. However, such approach may result in the loss of information about temporal dynamics, which can potentially contain prognostic value. Therefore, there is a need to implement more advanced time-series analysis techniques. Previous studies have reported reduced HR variance and entropy in nonsurvivors after TBI [61, 91]. Sykora et al. noted that, given the consistent results concerning the impact of the ANS on outcomes after TBI, the temporal evolution of ANS activity during the acute phase should be explored [77] Similarly, Froese et al. highlighted the importance of individual temporal trends in ANS metrics and their dynamic relationship with cerebrovascular reactivity [26]. In our previous study, we demonstrated that analysis of simultaneous transients between cerebral hemodynamics and the ANS using windowed time-lagged cross-correlation matrices has the potential for outcome prediction after TBI [82].

Our results suggest that including the correlation between ANS and cerebral hemodynamics may improve the discriminative ability of the PSH risk model. The most important associations were found between HR–ICP and HR–PRx. The analysis of the aggregated logistic regression coefficients for ZCR_HR-PRx_ and ZCR_HR-ICP_ indicates that greater instability was associated with a higher likelihood of PSH. Although these findings suggest that increased variability between ANS and cerebral hemodynamics could be a pathological factor, we acknowledge that due to a limited number of observations, these results should be interpreted with caution. The significance of HR–PRx relationship may derive from the influence of ANS on cerebral blood flow (CBF) regulation [2, 10]. Studies have shown that pharmacological blockade of the ANS alters CBF, suggesting an important autonomic role in CBF control [60, 96]. The association between autonomic dysfunction and cerebral autoregulation has been studied in both the acute and chronic phases of neurological conditions [14, 49, 50, 69, 83]. The significance of HR–ICP relationship reflects the impact of TBI on systemic circulation. TBI often leads to brain bleeding or swelling, causing an increase in ICP and a corresponding reduction in the CPP and CBF. This, in turn, may activate the ANS response, involving both parasympathetic and sympathetic branches. Reductions in CBF stimulate the release of vasoactive substances that induce arterial dilation, a process controlled by the parasympathetic branch of the ANS. Conversely, the sympathetic branch modulates ABP and HR to enhance CPP [23]. However, this mechanism assumes an intact ANS. In patients at risk for PSH, early pathological changes in the ANS may disrupt this balance, affecting the dynamic relationship between HR and the ICP.

PSH can occur at any stage following brain injury, and the exact time for its development remains under investigation. Some studies have reported that most clinical features appear within two weeks [15, 55], whereas others suggest that symptoms typically emerge five to seven days after injury [76]. In our cohort of patients, PSH was diagnosed between days nine and fifteen, while PSH risk model was based on observations made within the first 72 h after trauma, before the onset of specific alarming symptoms. The acute-phase response following head injury is driven by elevated levels of cytokines such as interleukins [16]. It has been previously reported that changes in physiological parameters within the first 72 h of TBI can predict mortality [22], as vasogenic and cytotoxic edema, proportional to the severity of trauma, peaks at approximately 72 h [56, 70]. Identifying patients at risk of PSH during the early period could enable timely interventions, improve patient outcomes, and reduce the severity of PSH-related complications. The temporal profile of significance for ANS-cerebral hemodynamics correlation indicated that within the 72-h period, the first three hours are the most important for PSH risk assessment. However, it should be acknowledged that although dysautonomia observed in TBI patients may outweigh the transient effects of concomitant factors, such as drugs or mechanical respiration, they may still affect the results of time-dependent feature importance analysis, especially during the initial hours after patient admission, when physiological parameters have not yet stabilized. Moreover, the absence of importance observed for the correlation between ANS and cerebral hemodynamics in the rest of the period may be attributed to the methodological limitations, as logistic regression primarily captures linear relationships and may not fully characterize the complexity of this interaction.

The clinical manifestations of PSH include tachycardia, tachypnea, hypertension, hyperthermia, hyperhidrosis, and posturing [5, 24]. Despite the establishment of the PSH-AM scoring system through international consensus [5], the diagnosis of PSH remains challenging due to the probabilistic character of this tool. PSH shares overlapping manifestations with other serious conditions, such as hydrocephalus, hypoxia, pulmonary embolism, and epileptic seizures [97]. Furthermore, treatments commonly administered to patients with severe TBI to minimize secondary brain damage, such as sedation and analgesia, can mask the manifestation of some symptoms [94]. As highlighted in previous studies, an elevated temperature may initially be attributed to inflammation or sepsis, but the early onset of fever following TBI may reflect impending autonomic dysfunction [33]. Given the complexity of PSH presentations, the development of alternative PSH risk models is needed.

There are several hypotheses regarding the mechanism by which brain injuries lead to the development of PSH [97]. One suggestion is that autonomic dysfunction may be triggered by diencephalic–mesencephalic dysfunction or disconnection [46]. The inhibitory ratio model of PSH proposes that functional disconnection within the central autonomic network results in the loss of greater control over the brainstem and spinal cord autonomic centers, rendering them hyperexcitable to afferent stimulation [4]. Another potential mechanism involves the neuroendocrine axis. Activation of the hypothalamic–pituitary–adrenal axis increases circulating catecholamine levels, which may serve as a protective mechanism to maintain CPP in the presence of increased ICP. However, this activation also has several adverse effects, including sympathetic excitation [30, 94].

We found that specific lesions on CT (tSAH, SDH, tICH) were among the most important features for PSH risk assessment. Recent research using magnetic resonance imaging (MRI) to explore the link between structural damage to the central autonomic network and PSH has shown that in a multivariable model adjusted for age, sex, and GCS, susceptibility-weighted imaging lesions in the corpus callosum and medial temporal lobes serve as independent imaging predictors of PSH [64]. Additionally, patients with PSH tend to have more deep lesions on cranial MRI [46], more focal lesions on cranial CT [25], DAI [47], intraventricular hemorrhage/subarachnoid hemorrhage, and complete cisternal effacement [63]. In our study, we did not specify the exact location of the lesions, which is an additional limitation of the study. Further research on the relationship between PSH risk and the localization of changes in cranial CT images is needed.

In our study, we included only the basic blood cell markers: Hb and WBC. Brain trauma is associated with increased serum catecholamine and cortisol levels, which release neutrophil stores and cause a decrease in the egress of neutrophils from the circulation [71]. The release of interleukins may contribute to an increase in the WBC count [53]. It has been hypothesized that WBCs may form aggregates, which, by adhering to one another, can occlude the microcirculation, leading to further ischemic damage [71, 74]. We found that the WBC count at admission was significantly greater in patients at risk of PSH than in patients not at risk, suggesting a more active systemic neuroinflammatory state in these patients. Hb transports more than 95% of O_2_ in the blood, and each unit of decrease in Hb reduces its oxygen-carrying capacity, resulting in anemic hypoxia [29]. The relationship between Hb levels and CBF has been studied in both animal models and humans, with findings indicating that CBF is inversely correlated with Hb concentration. This inverse relationship is interpreted as a homeostatic response of CBF to changes in CO_2_ concentration aimed at maintaining an adequate oxygen supply [36, 52]. A study by Von Kummer et al. revealed that an increase in CBF was associated with compensatory vasodilation of small distal cerebral arteries in response to low Hb levels to maintain oxygenation [88]. However, Hb is often a surrogate of injury severity, with more severely injured patients receiving large amounts of fluids, which results in low Hb levels [80]. Recent randomized clinical trials have also not shown a conclusive benefit of blood transfusion following TBI [78, 81], further emphasizing the complicated interplay between Hb levels and outcomes following TBI. The potential role of other serum and cerebrospinal fluid biomarkers, as well as genetic factors, in PSH development has not yet been studied. However, their role in predicting secondary complications after brain trauma has been demonstrated [43]. One of the most recent studies on early clinical descriptors of secondary events suggests that first-day ICU admission data on glucose and brain biomarkers, including S100B, neuron-specific enolase, neurofilament light, tau, ubiquitin carboxy-terminal hydrolase L1, and glial fibrillary acidic protein, may reliably distinguish disease trajectories in the ICU [3]. Therefore, further models of PSH risk involving a wider panel of biomolecular and genetic factors are needed [18, 39, 95].

This study has potential limitations. The models are based on a retrospective study with a limited number of patients, introducing inherent biases that may affect the representativeness of the cohort and the generalizability of the findings. We acknowledge that the main limitation of the proposed model is the lack of external validation. This is due to the data required to construct the model, which necessitates the integration of three types of information: the PSH-AM score for each patient, high-resolution data continuously monitored for 72 h—starting within the first 24 h after brain trauma—and clinical metadata, including CT characteristics. Nevertheless, an external validation of clinical prediction models represents an inherent and important part in their development, as in order to generalize, the input data needs to be representative [40]. In our study, to ensure generalizability, stratified five-fold cross-validation was applied, maintaining a consistent class distribution across training and validation sets. It has been demonstrated that implementing a nested cross-validation approach offers a nearly unbiased estimate of the true error [86]. The patients in this cohort were treated according to the guidelines in place at the time (Brain Trauma [9, 13]). However, the drugs administered (such as osmotic agents, vasopressors, sedative drugs), as well as mechanical ventilation, may have influenced the ANS response. Previous studies have shown that opioid drugs may impact baroreflex [42, 75]. On the other hand, a recent study on the impact of sedative and vasopressor agents on cerebrovascular reactivity indicated that commonly administered agents with incremental dosing changes have no clinically significant influence on cerebrovascular reactivity in TBI patients [27]. Our patients were intubated and mechanically ventilated during data collection. Mechanical ventilation has been shown to induce periodic changes in intrathoracic pressure, modulating venous return, which may further alter cardiovascular and cerebrovascular interactions [21, 26, 37, 65]. Although dysautonomia observed in TBI patients is likely to outweigh the transient effects of these factors, they may still affect the results of time-dependent feature importance analysis. Additionally, the response of the autonomic system to shock may differ between patients with isolated head trauma and those with polytrauma [54]. The obtained results need to be validated and repeated in a larger, more diverse cohort of patients to assess the real performance of the provided methods. Furthermore, the GOS should not be used at hospital discharge, as it requires the return to normal routines to be properly assessed. However, these data were unavailable to us, which is why we used hospital discharge as a surrogate.

## Conclusions

Including the correlation between ANS activity and cerebral hemodynamics estimated within 72 h after TBI may improve the performance of the clinical characteristics-based PSH risk model. The most important associations for further development of PSH syndrome were observed between HR–ICP and HR–PRx. Further studies in larger cohorts are necessary to validate these findings.

## Supplementary Information

Below is the link to the electronic supplementary material.

Supplementary file1 (DOCX 25 KB)

## Data Availability

The data that support the findings of this study are available upon reasonable request.
