# Supplementary material for: Paroxysmal sympathetic hyperactivity risk modeling based on transients in time series describing the autonomic nervous system and cerebral hemodynamics
Source: Acta Neurochir (Wien). 2025 May 30;167(1):158. doi: 10.1007/s00701-025-06566-9 (PMC12125141; doi:10.1007/s00701-025-06566-9)
Supplement: Supplementary file 1 — Supplementary file1 (DOCX 25 KB) [file 701_2025_6566_MOESM1_ESM.docx]

**SUPPLEMENTARY DATA**

**Paroxysmal Sympathetic Hyperactivity risk modeling based on transients in time series describing the autonomic nervous system and cerebral hemodynamics**

Mikołaj Najda^1^, Cyprian Mataczyński^2^, Małgorzata Burzyńska^3^, Magdalena Kasprowicz^4^, Jarosław Kędziora^3^, Emma Hammarlund^5^, Eric P Thelin^5,6^, Agnieszka Uryga^4#^

^1^ Maastricht University, Institute of Data Science, Maastricht, Limburg, The Netherlands

^2^ Wroclaw University of Science and Technology, Faculty of Information and Communication Technology, Wroclaw, Poland

^3^ Wroclaw Medical University, Faculty of Medicine, Clinical Department of Anesthesiology and Intensive Therapy, Wroclaw, Poland

^4^ Wroclaw University of Science and Technology, Department of Biomedical Engineering, Wroclaw, Poland

^5^ Medical Unit Neurology, Karolinska University Hospital, Stockholm, Sweden

^6^ Department of Clinical Neuroscience, Karolinska Institutet, Stockholm, Sweden

#Corresponding author:

Agnieszka Uryga

Department of Biomedical Engineering, Wroclaw University of Science and Technology,

Wybrzeze Wyspianskiego 27,

50-370 Wroclaw, Poland

e-mail: agnieszka.uryga@pwr.edu.pl

tel. +48 71 320 46 65

**Supplementary Table 1.** Evaluation of paroxysmal sympathetic hyperactivity (PSH) based on [1] [2].

| **Clinical Feature Scale (CSF)** | | | | | |
| --- | --- | --- | --- | --- | --- |
|  | 0 | 1 | 2 | 3 | Score |
| Heart rate | <100 | 100-119 | 120-139 | >=140 | 2 |
| Respiratory rate | <18 | 18-23 | 24-29 | >= 30 | 2 |
| Systolic blood pressure | <140 | 140-159 | 160-179 | >= 180 | 3 |
| Temperature | <37 | 37-37.9 | 38-38.9 | >= 39 | 2 |
| Sweating | Nil | Mild | Moderate | Severe | 3 |
| Posturing during episodes | Nil | Mild | Moderate | Severe | 1 |
| **CSF subtotal** | | | | | |
| **Diagnosis Likelihood Tool (DLT) (Score 1 point for each feature present)** | | | | | |
| Clinical features occur simultaneously | | | | | |
| Episodes are paroxysmal in nature | | | | | |
| Sympathetic over-reactivity to normally non-painful stimuli | | | | | |
| Features persist >= 3 consecutive days | | | | | |
| Features persist >= 2 weeks post brain injury | | | | | |
| Features persist despite treatment of alternative differential diagnosis | | | | | |
| Medication administered to decrease sympathetic features | | | | | |
| >= 2 episodes daily | | | | | |
| Absence of parasympathetic features during episodes | | | | | |
| Absence of other presumed cause of features | | | | | |
| Antecedent-acquired brain injury | | | | | |
| **DLT subtotal** | | | | | |
| **Combined total (CSF + DLT)** | | | | | |
| **PSH diagnostic likelihood** | | | Unlikely | <8 | |
|  |  |  | Possible | 8-16 | |
|  |  |  | Probable | >17 | |

[1] Baguley IJ, Perkes IE, Fernandez-Ortega J-F, Rabinstein AA, Dolce G, Hendricks HT, for the Consensus Working Group. Paroxysmal sympathetic hyperactivity after acquired brain injury: consensus on conceptual definition, nomenclature, and diagnostic criteria. J Neurotrauma 2014;31:1515–1520.

[2] Monteiro FB, Fonseca RC, Mendes R. Paroxysmal Sympathetic Hyperactivity: An Old but Unrecognized Condition. Eur J Case Rep Intern Med. 2017 Apr 27;4(3):000562.

**Supplementary Table 2.** TRIPOD checklist, according to: https://www.tripod-statement.org/

| **Section/Topic** | **I** | **Checklist Item** | **Page** |
| --- | --- | --- | --- |
| **Title and abstract** | | | |
| Title | 1 | Identify the study as developing and/or validating a multivariable prediction model, the target population, and the outcome to be predicted. | 1 |
| Abstract | 2 | Provide a summary of objectives, study design, setting, participants, sample size, predictors, outcome, statistical analysis, results, and conclusions. | 2 |
| **Introduction** | | | |
| Background and objectives | 3a | Explain the medical context (including whether diagnostic or prognostic) and rationale for developing or validating the multivariable prediction model, including references to existing models. | 3-4 |
|  | 3b | Specify the objectives, including whether the study describes the development or validation of the model or both. | 4 |
| **Methods** | | | |
| Source of data | 4a | Describe the study design or source of data (e.g., randomized trial, cohort, or registry data), separately for the development and validation data sets, if applicable. | 5 |
|  | 4b | Specify the key study dates, including start of accrual; end of accrual; and, if applicable, end of follow-up. | 5 |
| Participants | 5a | Specify key elements of the study setting (e.g., primary care, secondary care, general population) including number and location of centres. | 5-6 |
|  | 5b | Describe eligibility criteria for participants. | 5 (Fig.1) |
|  | 5c | Give details of treatments received, if relevant. | 5-6 |
| Outcome | 6a | Clearly define the outcome that is predicted by the prediction model, including how and when assessed. | 7 |
|  | 6b | Report any actions to blind assessment of the outcome to be predicted. | NA |
| Predictors | 7a | Clearly define all predictors used in developing or validating the multivariable prediction model, including how and when they were measured. | 8 |
|  | 7b | Report any actions to blind assessment of predictors for the outcome and other predictors. | NA |
| Sample size | 8 | Explain how the study size was arrived at. | 5 (Fig.1) |
| Missing data | 9 | Describe how missing data were handled (e.g., complete-case analysis, single imputation, multiple imputation) with details of any imputation method. | 8 |
| Statistical analysis methods | 10a | Describe how predictors were handled in the analyses. | 9 |
|  | 10b | Specify type of model, all model-building procedures (including any predictor selection), and method for internal validation. | 9-10 |
|  | 10d | Specify all measures used to assess model performance and, if relevant, to compare multiple models. | 9-10 |
| Risk groups | 11 | Provide details on how risk groups were created, if done. | 7 |
| **Results** | | | |
| Participants | 13a | Describe the flow of participants through the study, including the number of participants with and without the outcome and, if applicable, a summary of the follow-up time. A diagram may be helpful. | 5 (Fig.1) |
|  | 13b | Describe the characteristics of the participants (basic demographics, clinical features, available predictors), including the number of participants with missing data for predictors and outcome. | 10 (Tab.1) |
| Model development | 14a | Specify the number of participants and outcome events in each analysis. | 10 |
|  | 14b | If done, report the unadjusted association between each candidate predictor and outcome. | 10 (Tab.1) |
| Model specification | 15a | Present the full prediction model to allow predictions for individuals (i.e., all regression coefficients, and model intercept or baseline survival at a given time point). | 10-12 |
|  | 15b | Explain how to the use the prediction model. | 10-12 |
| Model performance | 16 | Report performance measures (with CIs) for the prediction model. | 10-12 |
| **Discussion** | | | |
| Limitations | 18 | Discuss any limitations of the study (such as nonrepresentative sample, few events per predictor, missing data). | 18 |
| Interpretation | 19b | Give an overall interpretation of the results, considering objectives, limitations, and results from similar studies, and other relevant evidence. | 12-17 |
| Implications | 20 | Discuss the potential clinical use of the model and implications for future research. | 13-18 |
| **Other information** | | | |
| Supplementary information | 21 | Provide information about the availability of supplementary resources, such as study protocol, Web calculator, and data sets. | Supp. Data |
| Funding | 22 | Give the source of funding and the role of the funders for the present study. | 19 |
